# Early discontinuation of long-acting reversible contraceptives methods and its associated factors in Hosanna town, central Ethiopia: a cross-sectional study

**DOI:** 10.1038/s41598-024-61648-5

**Published:** 2024-05-23

**Authors:** Amanuel Defar Bande, Tilahun Bayene Handiso, Habtamu Wude Hanjelo, Belayneh Hamdela Jena

**Affiliations:** 1 Hadiya Zone Health Bureau, Hossana, Ethiopia; 2 Department of Epidemiology, School of Public Health, College of Medicine and Health Sciences, Wachemo University, Hossana, Ethiopia; 3 Department of Health Service Management, School of Public Health, College of Medicine and Health Sciences, Wachemo University, Hossana, Ethiopia

**Keywords:** Long-acting reversible contraceptive, Early discontinuation, Factors, Ethiopia, Health care, Risk factors

## Abstract

Long-acting reversible contraceptive (LARC) method use is an ideal strategy for longer protection against unintended pregnancies, unsafe abortions, maternal morbidities, and mortalities related to pregnancies and childbirth. Despite low utilization of LARC methods in Ethiopia, early discontinuation remains a problem. This study aimed to assess prevalence of early discontinuation of LARC methods and associated factors in Hossana town. A community-based cross-sectional study was conducted among 433 adult women of reproductive age who had a history of LARC use. Logistic regression model was considered for the analysis. Proportion of LARC methods discontinuation within one year was 24.5%, 95% CI (20.6, 26.8%). Women whose age ≥ 30 years (AOR = 3.16, 95% CI: 1.27, 7.89), who had < 3 live children (AOR = 5.17, 95% CI 2.30, 11.61), who had a desire for pregnancy (AOR = 2.35, 95% CI 1.14, 4.85), who did not get pre-insertion counseling on the benefits of LARC methods (AOR = 1.79, 95% CI 1.01, 3.21) and who experienced side effects (AOR = 3.63, 95% CI 2.07, 6.38) were more likely to discontinue LARC methods early than their counterparts. Nearly one-fourth of clients discontinued using the LARC methods within the first year of insertion, highlighting the need to promote longer use for improved protection and success of family planning programs.

## Introduction

Long-acting reversible contraception is a method of birth control that includes subdermal implants (Implanon and Jadelle) and intrauterine contraceptive devices (IUCDs)^1^. The subdermal implants prevent unintended (mistimed and unwanted) pregnancies for at least three years, and the IUCDs protect for up to ten years. The long-acting reversible contraceptive (LARC) methods are effective, with a clinical failure rate of < 1% and fertility returning soon after removal^1,2^. The LARC methods have greater efficacy than short-acting contraceptive methods and are associated with lower rates of unwanted pregnancy^1–3^. Globally, about 15% of women use LARC methods, and some 3% use them in sub-Saharan Africa^4,5^. However, discontinuation of these effective methods of contraception is a universal problem, although rates vary widely by population and country^6,7^.

Long-acting reversible contraceptive method discontinuation is defined as stopping the use of LARC methods (Implants or IUCD) by women for any reason before the appropriate date^1,3,4,8^. Early discontinuation of LARC methods refers to the removal of the methods by health professionals and clients, stopping their use within one year of insertion^1,9,10^. Long-acting reversible contraceptive discontinuation has become a public health problem in developing countries, including Ethiopia, which accounted for 18–63%, and the majority of these discontinuations are among women who are still in need of contraception. Consequently, every year, about 182 million unplanned pregnancies occur in the world, which leads to high fertility rates and maternal deaths^2,11^.

Every day in 2017, approximately 810 women died from preventable (37–90%) causes related to pregnancy and childbirth, such as postpartum hemorrhage, preeclampsia/eclampsia, and sepsis^12–14^. The maternal mortality ratio is higher for low-income countries, especially Sub-Saharan Africa (66%), than in high-income countries, with 94% occurring in low and lower-middle-income countries^12,15^. Ethiopia, with a MMR of 412 per 100,000 live births, is one of the countries that contributes to the highest maternal mortality rates in the world^12,16^. Among others, part of these maternal deaths (~ 44%) can be averted by improved utilization of modern contraceptive methods, especially LARC methods, since they give longer protection from unintended pregnancies and pregnancy-related maternal morbidities and mortalities^17^. Globally, it is estimated that about 87 million unwanted pregnancies each year can be prevented by effective utilization of LARC methods^2^. Continuous use of LARC methods also prevents pregnancy in women who are medically unfit for pregnancy until their condition has improved. However, discontinuation of LARC methods remains a problem, especially in developing countries^7,18^. In Ethiopia, the pooled prevalence of LARC method discontinuation was reported to be 36.9%^17^.

Previous community-based studies reported a prevalence of early discontinuation of LARC methods that ranges from 10.3 to 22.5%^9,10^. Health facility-based studies reported a prevalence of early LARC method discontinuation that ranges from 57 to 69.8%^6,19^. In Ethiopia, about 35% of LARC method users discontinued using the methods within one year of insertion^8^. However, the studies vary in population, setting, study design, and sample size. Likewise, different factors associated with early discontinuation of LARC methods were identified, including but not limited to age, marital status, education, desire for pregnancy, previous experience of using contraceptive methods, husband approval, pre-insertion counseling, and side effects related to LARC methods^6,9,20–22^. These factors vary from place to place, as contraceptive utilization depends on socio-cultural issues, the accessibility and availability of the methods, and the existence of trained health professionals who provide the LARC methods.

Despite improvements in the availability and utilization of family planning in Ethiopia, early discontinuation of LARC is becoming a major problem that contributes to pregnancy-related maternal morbidities and mortalities^8^. Ethiopia shared the United Nations' agenda for sustainable development to reduce maternal mortality to below 70 per 100,000 live births by 2030^23^. The Ethiopian Ministry of Health has undertaken a LARCs scale-up initiative aimed at expanding access to and enhancing use of LARCs at the community level to reduce maternal mortality through trained health extension workers and health workers at all levels of the health care system^20,24^. In Ethiopia, utilization of LARC methods was low; 8% of married and 11% of sexually active unmarried women use implants, while 2% of married and 1% of sexually active unmarried women use IUCD. About 11% and 13% of reproductive-age women discontinue using implants and IUCD in the first year of insertion, respectively^16^. Evidence regarding the prevalence of early discontinuation of LARC methods and its associated factors, especially among adult reproductive-age women, in the study areas is scarce. Therefore, the aim of this study was to assess the prevalence and factors associated with the early discontinuation of LARC methods in Hosanna Town, central Ethiopia.

## Methods and materials

### Study area and period

Hossana town is located in Hadiya Zone in central Ethiopia, at a distance of 232 km from Addis Ababa, the capital city of Ethiopia. The town has six administrative kebeles with a total population of 115,393 (according to the 2007 Ethiopia census projection), of which 58,850 (51%) were females; 26,887 belong to the reproductive age group (15–49 years). The town has 1 comprehensive specialized hospital, 3 health centers, 6 health extension sites, 21 medium private clinics, and 15 private primary clinics. All governmental health institutions were providing long-acting family planning services.

The study was conducted in Hosanna town from July 26 to September 30, 2022.

### Study design and population

A community-based cross-sectional study was conducted among adult women of reproductive age (18–49 years) who had ever used long-acting reversible contraceptive methods.

### Eligibility criteria

Adult women who were in reproductive age and had ever used long-acting reversible contraceptive methods within the last five years from the date of data collection were included. However, women who were using long-acting reversible contraceptive methods but whose duration of use was less than one year were excluded, as this study defined discontinuation within one completed year of starting using the methods. Additionally, those women who were sick and unable to respond were excluded.

### Sample size and sampling procedure

The total sample size of 433 was estimated using Epi Info software version 7.2.2.6 by considering the following assumptions: the prevalence of LARC discontinuation of 10.3% taken from a previous study conducted in Sidama Regional State, a 95% confidence level, 3% marginal error, and a non-response rate of 10%^9^. The calculated sample size was proportionally allocated to each of the six Kebeles based on the previous one-year LARC use by women, which was obtained by referring to the family planning registration books of health facilities. In each kebele, the average number of women who used LARC in one year (April 1/2021–30/2022) was as follows: Heto (210), Jello naremo (254), Sech duna (230), Lichamba (194), Areda (243), and Bobicho (251). Eligible study participants from each kebele were included in the study by simple random sampling (random number generator) using the list of family folders (sample frame) that were obtained from the health extension workers documents. When two or more participants exist in the same household, a simple random sampling method (the lottery method) is applied. In cases where eligible study participants were absent at the time of the visit, a maximum of three visits were made. When the participant is still absent, another participant is considered from the sample frame.

### Variables

#### Dependent variable

Early discontinuation of LARC methods. It was categorized as ‘yes’ if clients discontinued using LARC methods within 12 months or ‘no’ if clients used the LARC methods for at least 12 months. For the analysis, ‘yes’ is coded as ‘1’ and ‘no’ is coded as ‘0’.

#### Independent variables

The independent variables include: (I) sociodemographic characteristics such as age, religion, occupation, marital status, and education. (II) Obstetric factors such as parity, abortion, and number of children. (III) Social factors such as husband opposition, husband involvement, source of information, and neighbors influence. (IV) method-related factors such as fear of side effects, desire for pregnancy, shifting or switching of contraception, past contraceptive utilization, and weight gain. (V) Facility and service provider factors such as pre-insertion counseling, informed choice, and follow-up.

### Operational definitions

*Long-acting reversible contraceptives* are methods (sub-dermal implants and intrauterine devices) that can prevent pregnancy for 3–10 years without requiring user action and return fertility soon after removal^6^.

*Early discontinuation of long-acting contraceptive methods* refers to the removal of the LARC methods by health professionals and clients stopping their use within one year of insertion^1,9,10^.

*Side effect:* refers to women developing at least one side effect after implants or IUCD insertion like menstrual disruption, insertion arm pain, weight gain or loss, headache, acne, and others that leads to the removal of the method.

*Pre-insertion counseling:* refers to getting advice and having discussions with contraceptive providers about benefits, side effects, and any concerns related to contraceptive methods before the insertion of a chosen method.

### Data collection procedures

A data collection tool was developed from various published literature^1,6,9,10^. The questionnaire was first prepared in English and translated to Amharic, then translated back to English to check consistency. Translations were done by experts in both languages. The Amharic version of the questionnaire was used for data collection. The data was collected using a pre-tested structured and semi-structured face-to-face interview questionnaire that contains four main parts. These are: socio-demographic characteristics; obstetric-related factors; contraceptive methods-related and facility-related variables; and service provider-related variables. Among the discontinuers, further information was required regarding the duration of LARC use and the reason for removal. The age, date of insertion of implants, or IUCD, and date of removal were asked during the data collection process. Four midwives who have a college diploma and two public health professionals who have a Bachelor degree were involved in data collection and supervision, respectively. One-day training was given for the data collectors and their supervisors on the purpose of the study and the overall data collection procedure.

### Quality control measures

The questionnaire was pre-tested one week before the actual data collection on 5% of LARC users’ women outside of the study area (Fonko town). Based on the pretest findings, necessary modifications such as skip and order were made to the questionnaire. Data collectors and supervisors were trained for one day. Interviewers were supervised at each site, and regular meetings were held between the data collectors and the supervisor. At the end of each data collection day, the questionnaire was reviewed and cross-checked for completeness and consistency by the investigator, and corrective discussion was undertaken. The data were edited and cleaned before analysis. Adjustment for potential confounding variables was done in multivariable model.

### Analysis

The collected data were checked, coded, and entered into Epi Data version 4.6 to minimize data entry errors. Then the data were exported to Statistical Package for Social Sciences (SPSS) version 21 for analysis. Descriptive statistics such as frequencies, percentages were done for categorical variables. For continuous variables, mean and standard deviation were calculated. For missing data, a complete case analysis was done. A binary logistic regression model was applied to identify factors associated with the early discontinuation of LARC. In binary logistic regression, potential factors associated with early discontinuation of LARC methods at *P* < 0.25 were recruited for the adjustment in the multivariable model. In multivariable logistic regression analysis, the adjusted odds ratio (AOR) with 95% CI was used to measure the strength of association, and variables with *P* < 0.05 were considered statistically significant. The model goodness of fit was checked by the Hosmer and Lemeshow test; when *P* > 0.05, the model fit the data reasonably better.

### Ethical approval

This study was carried out after the confirmation of international ethical guidelines for biomedical research involving human subjects^25^. The study was conducted after getting an approval letter from the Wachemo University Institutional Review Board (IRB), with a reference number [REF.No.WcU.RE.Dev.Di/315/2022]. A permission letter was communicated to concerned authorities before approaching study participants. Participants were informed about the purpose and objective of the study, and they have the right to discontinue or withdraw at any step of data collection. A written informed consent form was attached to the cover page of the questionnaire, and consent was obtained from the study participants by finger print or signature. The confidentiality of the participants was assured at every step of data processing by using codes rather than using personal identifiers such as name, village, etc. The authors had not accessed information that could identify individual participants during or after data collection, except analysing and interpreting aggregated participant information. All methods were carried out in accordance with the Declaration of Helsinki.

## Results

### Socio-demographic and reproductive characteristics

A total of 433 participants have responded to the questionnaire successfully, giving a response rate of 100%. Data was missed for husband education (8) and husband occupation (8). The mean age (± SD) of respondents was 28.26 (± 4.6) years. Among the participants, 339 (78.3%) were Hadiya in ethnicity, and 281 (64.9%) were protestant religion followers. More than half, 236 (54.5%), of the participants had given birth one to two times, and 182 (42%) of the respondents gave birth three to four times, and 15 (3.5%) of the respondents gave birth more than five times (Table 1).

**Table 1:** Socio-demographic and reproductive characteristics of participants in Hossana town, Hadiya zone, central Ethiopia, 2022.

Variables	Category	Frequency (%)
Marital status	Single	6 (1.4)
	Married	420 (97)
	Divorced/Widowed/	7 (1.6)
Religion	Protestants	281 (64.9)
	Orthodox	93 (21.5)
	Muslim	38 (8.8)
	Others*	21 (4.8)
Ethnicity	Hadiya	339 (78.3)
	Kambata	39 (9.0)
	Siltie	24 (5.5)
	Gurage	22 (5.1)
	Others**	9 (2.1)
Educational status	No formal education	338 (78.1)
	Primary (1–8 grades)	11 (2.5)
	Secondary (9–12 grades)	29 (6.7)
	Higher education	55 (12.7)
Occupation status	Housewife	226 (52.2)
	Government employee	65 (15)
	Private employee	73 (16.9)
	Merchant	69 (15.9)
Husband education	No formal education	220 (53.8)
	Primary (1–8 grades)	14 (3.2)
	Secondary (9–12 grades)	35 (8.1)
	higher education	151 (34.9)
Husband’s occupation	Government employee	163 (38.8)
	private employee	23 (5.5)
	Daily laborer	51 (12.1)
	Merchant	183 (43.6)
Monthly income	< 2000 ETB	33 (7.6)
	2000–3000 ETB	116 (26.8)
	3001–4000 ETB	177 (40.9)
	> 4000 ETB	107 (24.7)
Parity	1–2	236 (54.5)
	3–4	182 (42.0)
	5 and above	15 (3.5)
History of abortion	Yes	86 (19.9)
	No	347 (80.1)

*Others: Apolostic, Joba witness.

**Others: Amhara, Oromo, Tigrie, Wolayita.

### Contraceptive-related characteristics

Three hundred two (69.7%) of the study participants had ever used modern contraceptives before the LARC; out of them, 195 (45.0%) had used injectable followed by oral contraceptive pills 80 (18.5%) and implants 27 (6.2%) (Table 2).

**Table 2:** Contraceptive and service-related characteristics of participants among women who ever used LARC in Hosanna town, Hadiya zone, central Ethiopia, 2022.

Variables	Category	Frequency (%)
Any modern contraceptive used before LARC	Yes	302 (69.7)
	No	131 (93.03)
Type of contraceptive used before LARC	OCP	80 (18.5)
	Injectable	195 (45.0)
	Implanon	27 (6.2)
Type of LARC used	Implanon	310 (71.6)
	Jadelle	84 (19.4)
	IUCD	39 (9.0)
Place of insertion	Hospital	161 (37.2)
	Health center	272 (62.8)
Pre-insertion counseling on benefits of LARC	Yes	300 (69.3)
	No	133 (30.7)
Pre-insertion Counseling on side effect	Yes	292 (67.4)
	No	141 (32.6)
Appointment for checkup after insertion	Yes	346 (79.9)
	No	87 (20.1)
Counseling type	Individual	343 (79.2)
	With husband	64 (14.8)
	Mass	26 (6.0)
Ever experience side effect	Yes	231 (53.3)
	No	202 (46.7)
Who chose LARC	Own choice	275 (63.5)
	My husband	25 (5.8)
	Health professional	97 (22.4)
	Health extension	36 (8.3)
Reason for choosing the methods	Safety	108 (24.9)
	Long protection	273 (63.0)
	Can be removed at any time	45 (10.4)
	Immediate fertility return	7 (1.6)

### Prevalence of early discontinuation of LARC methods

Of a total of 433 adult women who ever used LARC methods, 106 (24.5%) (95% CI: 20.6–26.8%) had discontinued the methods within one year following the insertion. The median time of discontinuation was 14 months, with an interquartile range of 1 month. Implanon contributes for higher proportion of overall LARC methods early discontinuation, which is 78 (18%) followed by Jadelle, 20 (4.6%) and IUCD, 8 (1.9%). Regarding method specific discontinuation: of 310 implanon users, 78 (25.2%), of 84 Jadelle users, 20 (23.8%), and of 39 IUCD users, 8 (20.5%) had discontinued within one year of the insertion. The majority, 65 (61.3%) of the women, discontinued LARC methods between 6 and 12 months of use. The remaining 41 (38.7%) discontinued the methods within 6 months of use. Most, 61 (57.6%) of the participants discontinued due to side effects. About 42 (39.2%) discontinued due to desire for pregnancy and 3 (2.8%) due to preference for other methods. The most frequently raised complaints among women who faced side effects were menstrual irregularity, 31 (50.8%), headache, 14 (23%), insertion site pain, 8 (13.1%), weight gain, 3 (4.9%), and difficulty working, 5 (8.2%). The trend of discontinuation of LARC methods has shown an increasing pattern as the duration of months increases, especially after 12 months (Fig. 1).

### Factors associated with early discontinuation of LARC methods

In binary logistic regression analysis, women's age, number of live children, desire for pregnancy, contraceptive use before LARC methods, getting pre-insertion counseling on the benefits of LARCs, getting pre-insertion counseling on side effects, and experiencing side effects were identified as candidate variables for multivariable logistic regression. In multivariable logistic regression analysis, women's age, number of live children, desire for pregnancy, getting pre-insertion counseling on the benefits of LARCs, and experiencing side effects were independently associated with the early discontinuation of LARC methods.

In this study, the odds of early discontinuation of LARC methods was three times (AOR = 3.16, 95% CI: 1.27, 7.89) higher for women who belong to the age group of 30 years and above as compared to those in the age group of 20–24 years. The odds of early discontinuation of LARC methods was five times (AOR = 5.17, 95% CI: 2.30, 11.61) higher for women who had less than three live children than those who had greater than or equal to three live children. The odds of early discontinuation of LARC methods was twice (AOR = 2.35, 95% CI: 1.14, 4.85) higher for women who had a desire to be pregnant than for those who had no desire to be pregnant. The odds of early discontinuation of LARC methods was nearly twice (AOR = 1.79, 95% CI: 1.01, 3.21) higher for women who had not received pre-insertion counseling on the benefits of LARC methods as compared to those who had received pre-insertion counseling on the benefits. Likewise, the odds of early discontinuation of LARC methods was nearly four times (AOR = 3.63, 95% CI: 2.07, 6.38) higher for women who had experienced side effects as compared to those who had not experienced the side effects of a method used (Table 3).

**Table 3:** Multivariable logistic regression analysis for the factors associated with early discontinuation of LARC methods in Hosanna town, Hadiya Zone, central Ethiopia, 2022.

Variable	LARC discontinued		COR (95% CI)	AOR (95% CI)
	Yes (%)	No (%)		
Age in years				
20–24	23 (29.9)	54 (70.1)	1	1
25–29	60 (29.6)	143 (70.4)	0.99 (0.56, 1.75)	1.46 (0.78, 2.74)
≥ 30	23 (15)	130 (85)	0.42 (0.22, 0.80)**	3.16 (1.27, 7.89)*
Number of live children				
< 3	89 (36.5)	155 (63.5)	5.81 (3.31, 10.20)***	5.17 (2.30, 11.61)***
≥ 3	17 (9)	172 (91)	1	1
Desire for pregnancy				
Yes	88 (34.2)	169 (65.8)	4.57 (2.63, 7.93)***	2.35 (1.14, 4.85)*
No	18 (10.2)	158 (89.8)	1	1
Contraceptive used before LARCs				
Yes	64 (21.2)	238 (78.8)	0.57 (0.36, 0.90)*	0.78 (0.46, 1.32)
No	42 (32.1)	89 (67.9)	1	1
Counseled about the benefit of LARC				
Yes	84 (28)	216 (72)	1	1
No	22 (16.5)	111 (83.5)	1.96 (1.16, 3.31)*	1.79 (1.01, 3.21)*
Got pre-insertion counseling on side effects				
Yes	60 (20.5)	232 (79.5)	0.53 (0.34, 0.84)**	0.61 (0.36, 1.03)
No	46 (32.6)	95 (67.4)	1	1
Experienced side effects of LARC				
Yes	83 (35.9)	148 (64.1)	4.37 (2.62, 7.27)***	3.63 (2.07, 6.38)***
No	23 (11.4)	179 (88.6)	1	1

*AOR* Adjusted odds ratio; *COR* Crude odds ratio.

***P < 0.001; **P < 0.01; *P < 0.05; 1 = reference category.

## Discussion

This study has shown that the prevalence of early discontinuation of LARC methods was 24.5%. The early discontinuation of LARC methods was associated with older women’s age, number of live children, a desire for pregnancy, pre-insertion counseling on benefits, and experiencing side effects of the method used.

In this study, the observed prevalence of early discontinuation of LARC methods was substantial, as one-fourth of the users discontinued the method within one year of insertion. The result suggests the need to emphasize strategies that help improve the utilization of LARC methods for a longer duration, such as pre-insertion counseling on benefits, potential side effects, and an informed choice of the LARC methods. Clients who visit health facilities seeking contraceptive methods are often preoccupied by the methods that their significant others (husband, friends, or neighbors) suggest. Informed choices that are client-centered need to be promoted to decrease early discontinuation of LARC methods. The result of this study was consistent with the community-based study conducted in a neighborhood city in Butajira town, Southern Ethiopia, which was 22.5%^10^ and that of a prospective cohort study from urban health facilities in Ethiopia, which was 21.8%^1^. However, the finding of this study was higher than the reported prevalence from a cross-sectional study in the Sidama region of Ethiopia, which was 10.3%^9^. It is also higher than the findings from other parts of the world: Indonesia, which was 12%^26^, and the study conducted in 21 low-income countries, which was 9%^27^. The finding was lower than the proportion of early discontinuation of LARC methods reported from health facility-based studies; Hawassa City, Southern Ethiopia, which was 56.6%^6^, and Kampala-Uganda, which was 29%^28^. The observed differences in the proportions of early discontinuation of LARC methods might be due to variation in awareness of the benefits of the methods, myths and misconceptions related to the methods used, quality of counseling, socio-cultural conditions, desired number of children that the families wish to have, study population, sample size, and other contextual factors across the study settings^29^.

In this study, older women (age ≥ 30 years) were three times more likely to discontinue LARC methods as compared to younger women (20–24 years). This finding was contrary to the results of studies conducted in Kampala-Uganda^28^, Papua New Guinea^30^, and Nepal^21^, where younger women were more likely to early discontinue LARC methods than older women. However, the older age category and population included in the study vary across studies. In the Ugandan study, the population was those who had discontinuation, and the age was treated as a continuous variable. In Papua New Guinea, the population was composed of reproductive age women, and older age represented 45–49 years, and it was the reference category. In the Nepal study, the population was married women, and the older age group represented ≥ 35 years, compared with the age group of < 25 years. In our study, age ≥ 30 years (because of the very small cell for age > 35 years) was compared with the younger age group (20–24 years). Despite these variations in population and age categorization, the observed association in this study might be due to the fact that older women might have fewer reproductive years (as fertility usually declines after the age of 30 years), so they might wish to have additional children within the remaining childbearing years^31^. On the other side, those younger women might have their own reproductive plan or postpone their pregnancy due to further education and participation in income-generating activities. Furthermore, this study included those unmarried adult women so that they might have extended childbearing before marriage and use LARC methods without early discontinuation for longer protection of pregnancy.

Women who had fewer than three live children were five times more likely to early discontinue LARC methods than those who had three or more children. Women with a small number of children might have a desire for additional children, so they tend to early discontinue the method used^28^. Previous studies conducted in Kampala-Uganda^28^, Sidama, Ethiopia^9^, and Nepal^21^ reported related findings where women who have fewer live children were more likely to discontinue the LARC methods in the first year of use.

The finding of this study indicated that women who had a desire for pregnancy were twice more likely to early discontinue LARC methods than their counter parts. This finding was consistent with findings of previous studies in Ethiopia; Hawassa City^6^, and Butajira town^10^. Women who achieved their planned time for next pregnancy might have discontinued the LARC methods early and they also have used other methods before LARC methods so that they might have reached desired period of protection and discontinue the methods used.

Proper pre-insertion counseling about the benefits and potential side effects of contraceptive methods is an ideal strategy to promote the continuation of modern contraceptive methods, including LARC methods. In this study, women who had not received pre-insertion counseling on the benefits of LARC methods were twice more likely to early discontinue LARC methods than those who had received pre-insertion counseling on the benefits of LARC methods. The result was consistent with previous studies in Butajira town^10^, Hawassa city^6^, and the Sidama region^9^.

Likewise, women who experienced side effects were nearly four times more likely to early discontinue LARC methods than those who did not experience side effects. The result was consistent with the findings of Hawassa City^6^. Side effects of LARC methods in the first year of use are common, especially menstrual irregularity. Some clients still tolerate it, while others prefer to discontinue and switch to other methods or interrupt altogether^10,19,29^.

The findings have wider practical implications for the need to enhance pre-insertion counseling on the effectiveness and common side effects of LARC methods. Pre-insertion counseling enhances continued utilization of LARC methods and the success of family planning programs, including reducing wastage of LARC methods, as they have a longer duration of protection against unintended pregnancies and pregnancy-related maternal deaths^6,17^.

Despite the attempts made to reduce it this study might have limitations related to data collection. Recall bias related to remembering the date of insertion and removal of LARC methods might have occurred. A bias in reporting the age might have occurred, as we rely on the verbal reports of participants. Additionally, selection bias might have occurred due to the missing of some LARC method users that were not registered in the health extension workers records. Acknowledging these limitations, the findings from this community-based study can be generalized to populations with similar contexts.

## Conclusions

In this study, nearly one-fourth of clients discontinued using the LARC methods within the first year of the insertions, highlighting the need to promote longer use for improved protection and the success of family planning programs. Counselors should emphasize on pre-insertion counseling on the benefits and expected side effects of LARC methods. Capacity building trainings such as on the job and refreshment trainings may help achieve longer use of the LARC methods through proper counseling services.

## Data Availability

The raw materials that support the conclusions of this research will be available to researchers, who need the data to use for non-commercial purposes through requesting the corresponding author.

## Abbreviations

AOR: Adjusted odds ratio
CI: Confidence interval
COR: Crude odds ratio
IUCD: Intra-uterine contraceptive device
LARC: Long-acting reversible contraceptive
